# Supplementary material for: Serum and CSF metabolomics analysis shows Mediterranean Ketogenic Diet mitigates risk factors of Alzheimer’s disease
Source: NPJ Metab Health Dis. 2024 Jul 1;2:15. doi: 10.1038/s44324-024-00016-3 (PMC11216994; doi:10.1038/s44324-024-00016-3)
Supplement: Supplementary file 1 — Supplementary information [file 44324_2024_16_MOESM1_ESM.pdf]

Supplementary Figures

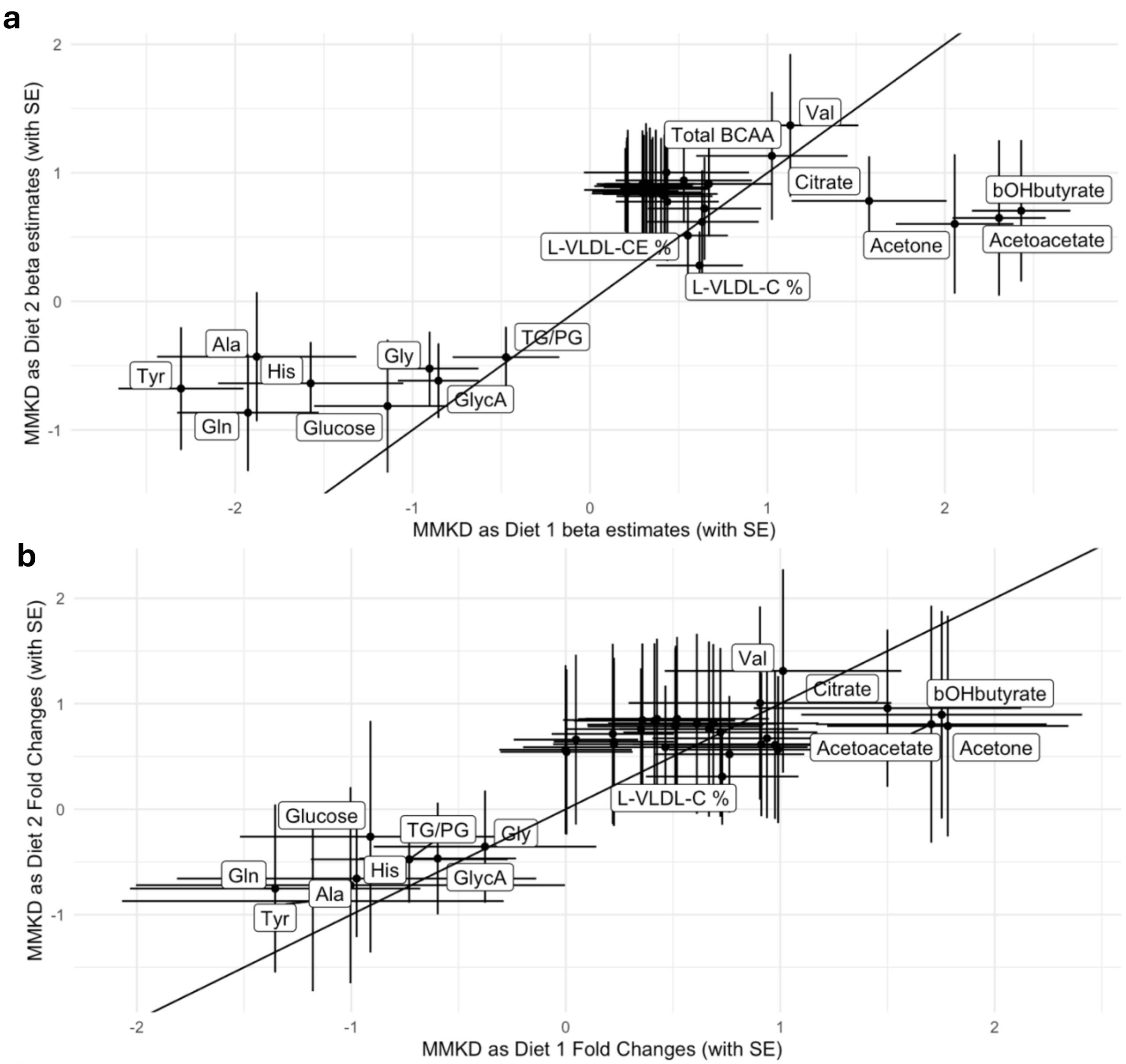

Supplementary Figure 1: Effect of diet order on serum metabolite beta estimates and fold changes. a. Beta estimates of serum metabolites found to significantly associate with the MMKD from the differential abundance analysis performed on the cohort with MMKD as their first diet (n = 11) on the x-axis and with MMKD as their second diet (n = 8) on the y-axis. Points are plotted with their standard errors for both analyses. b. Fold changes of metabolites found to significantly associate with the MMKD for the cohort with MMKD as their first diet on the x-axis and with MMKD as their first diet on the y-axis. Points are plotted with their standard errors for both analyses.

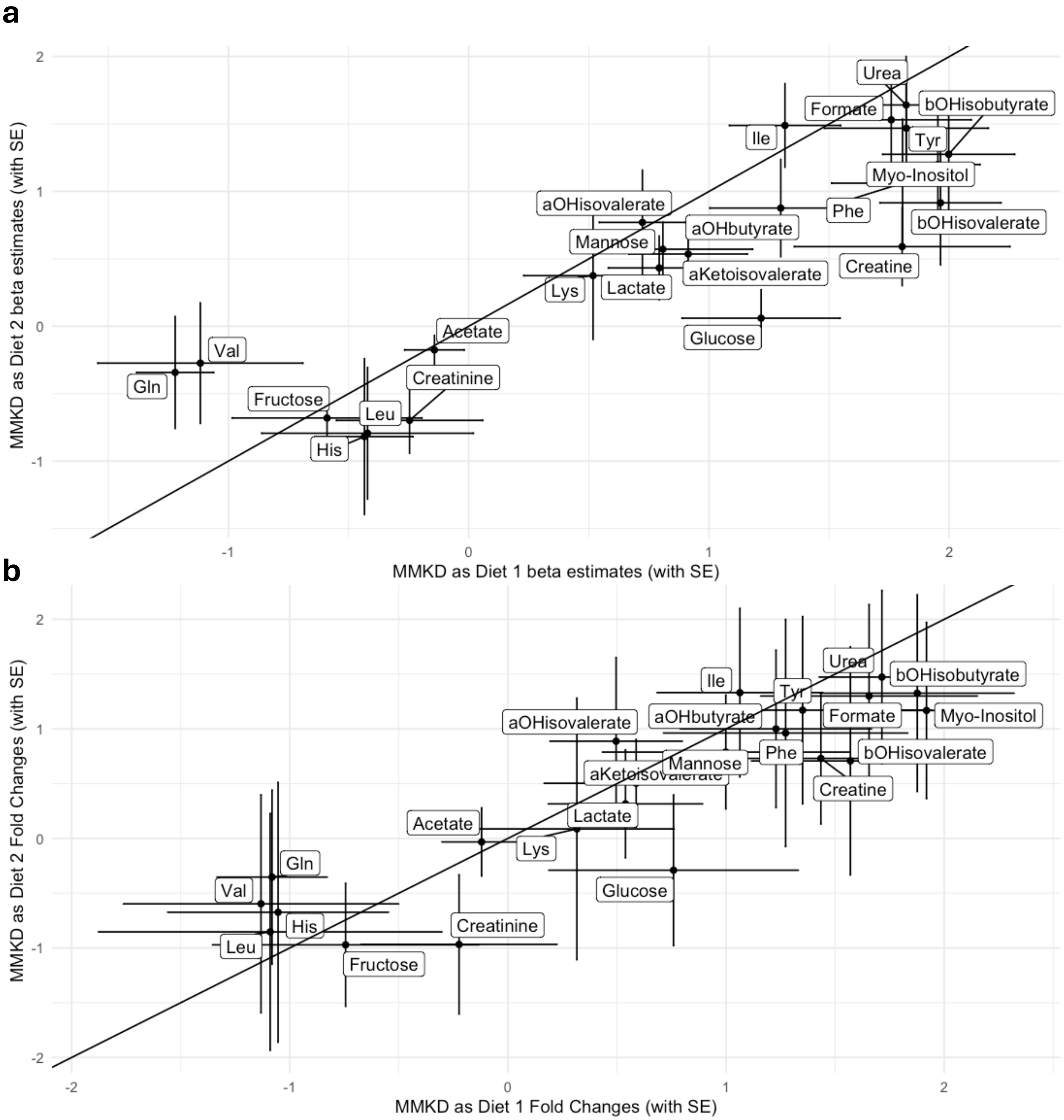

Supplementary Figure 2: Effect of diet order on CSF metabolite beta estimates and fold changes. a. Beta estimates of CSF metabolites found to significantly associate with the MMKD from the differential abundance analysis performed on the cohort with MMKD as their first diet (n = 11) on the x-axis and with MMKD as their second diet (n = 8) on the y-axis. Points are plotted with their standard errors for both analyses. b. Fold changes of metabolites found to significantly associate with the MMKD for the cohort with MMKD as their first diet on the x-axis and with MMKD as their first diet on the y-axis. Points are plotted with their standard errors for both analyses.

Supplementary Notes

Alzheimer's Gut Microbiome Project Consortium Members and Affiliations:

Rima Kaddurah-Daouk, Ph.D. (Duke University, Principal Investigator)
Alexandra Kueider-Paisley, Ph.D. (Duke University)
P. Murali Doraiswamy, M.B.B.S. (Duke University)
Colette Blach, M.S. (Duke University)
Arthur Moseley, Ph.D. (Duke University, PI)
Siamak Mahmoudiandehkhordi, Ph.D. (Duke University)
Leyla Schimmel, Ph.D. (Duke University)
Kathleen Welsh-Balmer, Ph.D. (Duke University)
Brenda Plassman, Ph.D. (Duke University)
Andrew Saykin, Psy.D. (Indiana University School of Medicine, PI)
Kwangsik Nho, Ph.D. (Indiana University School of Medicine, Co-I)
Shannon Risacher, Ph.D. (Indiana University School of Medicine)
Jared Brosch, M.D. (Indiana University School of Medicine)
Gabi Kastenmüller, Ph.D. (Helmholtz Zentrum München, PI)
Matthias Arnold, Ph.D. (Helmholtz Zentrum München, Co-I)
Xianlin Han, Ph.D. (University of Texas Health Science Center San Antonio)
Rebecca Baillie, Ph.D. (Rosa & Co., LLC)
Rob Knight, Ph.D. (University of California – San Diego, PI)
Pieter Dorrestein, Ph.D. (University of California – San Diego)
James Brewer (University of California – San Diego)
Emeran Mayer, M.D. (University of California – Los Angeles, PI)
Jennifer Labus (University of California – Los Angeles)
Pierre Baldi, Ph.D. (University of California – Los Angeles)
Arpana Gupta, Ph.D. (University of California – Los Angeles)
Oliver Fiehn, Ph.D. (West Coast Metabolomics Center, University of California - Davis)
Dinesh Barupal, Ph.D. (Icahn School of Medicine at Mount Sinai)
Peter Meikle, Ph.D. (Baker Heart and Diabetes Institute, PI)
Sarkis Mazmanian, Ph.D. (California Institute of Technology, PI)
Dan Rader, Ph.D. (University of Pennsylvania, PI)
Leslie Shaw, Ph.D. (University of Pennsylvania)
Cornelia van Duijin, Ph.D. (Oxford University, PI)
Najaf Amin (Oxford University)
Russ Swerdlow, M.D. (Kansas University Medical Center)
Laura Baker, Ph.D. (Wake Forest University)
Susan Craft (Wake Forest University)
David Bennett, M.D. (Rush University)
Ranga Krishnan, M.D. (Rush University)
Ali Keshavarzian, Ph.D. (Rush University)
Robin Vogt-Zuwala, Ph.D. (Rush University)
Klodian Dhana, Ph.D. (Rush University)
Arfan Ikram, M.D., Ph.D. (Erasmus University Medical Center, PI)
Thomas Hankemeier, Ph.D. (Leiden University Metabolomics Center, PI)
Ines Thiele, Ph.D. (National University of Ireland – Galway, PI)
Priyanka Baloni, Ph.D. (Purdue University)

Wei Jia, Ph.D. (University of Hawaii Cancer Center)
David Wishart, Ph.D. (The Metabolomics Innovation Centre Canada, PI)
Peter Würtz, Ph.D. (Nightingale Health)
Therese Koal, Ph.D. (Biocrates Life Sciences AG)
Anna Greenwood, Ph.D. (Sage Bionetworks)
Jan Krumsiek, Ph.D. (Weill Cornell Medicine, Co-I)
Karsten Suhre, Ph.D. (Weill Cornell Medicine - Qatar)
Matej Oresic (Orebro University)
Kamil Borkowski (University of California - Davis)
John Newman, Ph.D. (United States Department of Agriculture - Agricultural Research Service)
Ivan Hernandez, M.D. (State University of New York - Downstate, PI)
Tatania Foroud, Ph.D. (National Centralized Repository for Alzheimer's and other Dementias, PI)
Frank Sacks, M.D. (Harvard University, T.H. Chan School of Public Health)

Supplementary Tables

Supplementary Tables 1-5 have been uploaded to the following figshare:
https://doi.org/10.6084/m9.figshare.25607856.v1

Supplementary Methods

Joint Tensor Factorization algorithm

We adopt the model setting proposed in [1], which can be used to reduce the dimensions jointly between multiple temporal tensors formed by $\bar{T}_1$ and $\bar{T}_2$ through the objective function:

$$\min_{\lambda, a_{i,2}\xi} \sum_{i,j,t \in T_i} \left(\left(\bar{T}_1\right)_{ijt} - \lambda_1 a_i b_{ij} \xi_{it} \right)^2 + \sum_{i,j,t \in T_i} \left(\left(\bar{T}_2\right)_{ijt} - \lambda_2 a_{i,} b_{2j} \xi_2 t \right)^2 \tag{1}$$

Where $q_1 \lambda_1, \lambda_2$ are updated separately.

$$\begin{aligned}
\hat{a}_i^{(t+1)} &= \underset{a}{\operatorname{argmin}} \left[\sum_{j,t \in T_i} \left(b_{1j}^{(t)} \xi_1^{(t)} \lambda_1^{(t)} \right)^{(t)} + \left(b_{2j}^{(t)} \xi_{2t}^{(t)} \lambda_2^{(t)} \right)^2 \right\} a_i^2 \\
\text{a } & -2 \sum_{j,t \in T_i} \left\{ \left(\bar{T}_1\right)_{ijt} \lambda_1^{(t)} b_{1j}^{(t)} \xi_{1t}^{(t)} + \left(\bar{T}_2\right)_{ijt} \lambda_2^{(t)} b_2 j \xi_2^{(t)} s_2^{(t)} \right\} a \Bigg] \\
&= \frac{\sum_{j \cdot t \epsilon T_i} \left\{ (\bar{T})_{ij\tau} b_{1j}^{(t)} \xi_{1t}^{(t)} \lambda_1^{(t)} + \left(\bar{T}_2\right)_{ijt} b_{2j}^{(t)} \xi_{2\tau}^{(\tau)} \lambda_2^{(t)} \right\}}{\lambda_1^{(t)} \sum_{t \in T_i} \xi_{1t}^{(t)2} + \lambda_2^{(t)2} \sum_{t \in T_i} \xi_{2t}^{(t)2}} \\
\hat{\lambda}_1^{(t+1)} &= \arg \lambda_1 \min \left[\sum_{i,j,t \in T_i} \lambda_1^2 \left(a_i b_{1j} \xi_1 t\right)^2 - 2 \sum_{i,j \cdot t \in T_i} \left(\bar{T}_1\right)_{ijt} a_i b_{1j} \xi_1 \lambda_1 \right] \\
&= \frac{\sum_{i,t \in T_i} \left(\bar{T}_1\right)_{itt} a_{vi} b_{ij} \xi_{1t}}{\sum_{i,t \in T_i} \left(a_i \xi_{1t}\right)^2} \\
\hat{\lambda}_2^{(t+1)} &= \frac{\sum_{i,j=t \in T_i} \left(\bar{T}_2\right)_{ijt} a_i b_{2j} \xi_{2t}}{\sum_{i,j \in T_i} \left(a_i \xi_{2t}\right)^2} \\
\hat{b}_{(j)}^{(t+1)} &= \frac{\sum_{i,t \in T_i} a_i \xi_{1t} \left(\bar{T}_1\right)_{jit}}{\sum_{i,t \in \pi} \left(a_i \xi_1 t\right)^2}, \hat{b}_1^{(t+1)} = \hat{b}_1^{(t+1)} / \left\| \hat{b}^{(t+1)} \right\|_2 \\
\hat{b}_{2j}^{(t+1)} &= \frac{\sum_{i \in I_i} a_i \xi_{2t} \left(\bar{T}_2\right)_{ijt}}{\sum_{i,t \in T_i} \left(a_i \xi_{it}\right)^2}, \hat{b}_2^{(t+1)} = \hat{b}_2^{(t+1)} / \left\| \hat{b}_2^{(t+1)} \right\|_2
\end{aligned} \tag{2}$$

where a are subject loadings, b are feature loadings that can be used to identify features contributing to the beta analysis, $\xi^{(l)}(t)$ is the temporal loading that captures the shared temporal patterns among subjects and features, and λ quantifies the contribution of each component. For each b^1 and b^2 we define W^1 and W^2 respectively given by:

$$\begin{aligned}
\mathrm{W}^1 &= (\lambda_1 b^{1^T})^T \\
\mathrm{W}^2 &= (\lambda_2 b^{2^T})^T
\end{aligned} \tag{3}$$

The temporal covariance and correlations of all features across all input matrices are calculated from the final estimated matrices by:

$$\textit{temporal feature covariance matrix} = \begin{bmatrix} \boldsymbol{W_1} \\ \boldsymbol{W_2} \end{bmatrix} \begin{bmatrix} \boldsymbol{W_1} \\ \boldsymbol{W_2} \end{bmatrix}^T \quad (4)$$

Which provides the estimated relationships between the features across both input temporal tensors across time.

Supplementary References

1. Shi, P. *et al. Time-Informed Dimensionality Reduction for Longitudinal Microbiome Studies* en. July 2023. `https://www.biorxiv.org/content/10.1101/2023.07.26.550749v1`.
